# Controlling the drug-resistant tuberculosis epidemic in India: challenges and implications

**DOI:** 10.4178/epih.e2021022

**Published:** 2021-04-07

**Authors:** Aliabbas A. Husain, Andreas Kupz, Rajpal S. Kashyap

**Affiliations:** 1 Research Centre, DR. G.M. Taori Central India Institute of Medical Sciences (CIIMS), Nagpur, India; 2 Centre for Molecular Therapeutics, Australian Institute of Tropical Health and Medicine, James Cook University, Cairns, Australia

**Keywords:** Tuberculosis, India, Drug resistance, Diagnosis, Epidemics

## Abstract

India has a higher tuberculosis (TB) burden than any other country, accounting for an estimated one-fourth of the global burden. Drug-resistant tuberculosis (DR-TB) presents a major public health problem in India. Patients with DR-TB often require profound changes in their drug regimens, which are invariably linked to poor treatment adherence and sub-optimal treatment outcomes compared to drug-sensitive TB. The challenge of addressing DR-TB is critical for India, as India contributes over 27% of global DR-TB cases. In recent decades, India has been proactive in its battle against TB, even implementing a revised National Strategic Plan to eliminate TB by 2025. However, to achieve this ambitious goal, the country will need to take a multifaceted approach with respect to its management of DR-TB. Despite concerted efforts made by the National TB Elimination Program, India faces substantial challenges with regard to DR-TB care, especially in peripheral and resource-limited endemic zones. This article describes some of the major challenges associated with mitigating the growing DR-TB epidemic in India and their implications.

## INTRODUCTION

**Figure 1.**
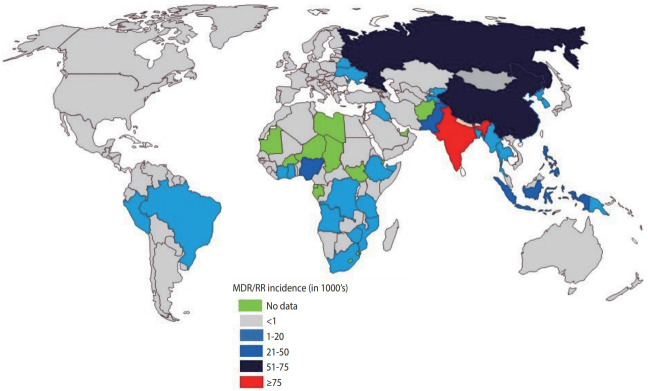
Global incidence of MDR/RR-TB (in thousands). India has the highest incidence burden of MDR/RR-TB (135,000), followed by China (73,000) and Russia (56,000). MDR, multidrug-resistant; RR, rifampicin-resistant; TB, tuberclosis. Source from: World Health Organization. Tuberculosis; 2020 [1].

India continues to have one of the highest rates of tuberculosis (TB) incidence and mortality of any country [1]. According to the India TB Report 2020, there were an estimated 2.69 million cases of TB in India, accounting for a quarter of all global TB cases [2]. Since its launch in 1997, the National Tuberculosis Elimination Program (NTEP) (formerly the Revised National Tuberculosis Control Program) has achieved considerable strides and success through its policy changes over time, with the aim of reducing the burden of TB in India (Table 1). The National Strategic Plan (NSP) is a regulatory framework rolled out by the government of India to guide TB-related stakeholders and key policy-makers, central and state authorities, and other health bodies in the elimination of TB. The NSP’s main objective is aligned with the World Health Organization (WHO) End Tuberculosis Strategy and the United Nations (UN) Sustainable Development Goals (SDGs) for the elimination of TB. In 2017, India launched its revised NSP (2017-2025) with the ambitious and challenging goal of eliminating TB by 2025, 5 years before the 2030 target set by the UN-SDG and WHO End Tuberculosis Strategy [2]. To achieve the objectives of the NSP, India must reduce TB cases by at least 10% every year, compared to the global target of 2%. The revised NSP includes several new recommendations including scaling up of rapid molecular diagnostic services to reduce the burden of drug-resistant tuberculosis (DR-TB) [2]. Despite significant efforts, the incidence of TB has been declining slowly (1.8%) with a substantial increase in the number of drug-resistant (DR) cases, which is estimated to comprise 27% of the world’s cases [3]. India, China, and Russia combined contribute more than half of the multidrugresistant tuberculosis (MDR-TB) cases globally (Figure 1) [1].

India started managing DR-TB through the Programmatic Management of Drug-resistant Tuberculosis (PMDT, formerly Directly-observed Treatment Short Course [DOTS Plus]) under the 2007 NTEP guidelines, which were released in 2010 and further updated in 2017 and 2019 [4]. In 2014, standards for TB care in India were released, which included comprehensive guidelines for the treatment and management of TB and DR-TB [5]. The current updated services of PMDT under the revised NSP include key policy revisions aligned with WHO recommendations for DR-TB care such as universal drug susceptibility testing (U-DST) for presumptive TB cases, scaling-up of cartridge-based nucleic acid amplification tests (CBNAAT), TrueNAT and line probe assay (LPA) services, advisory guidance for shorter/longer oral regimens for MDR and rifampicin-resistant (RR) cases, and decentralization of DR-TB services for better accessibility [4]. Despite significant strides and a stronger political commitment under the NSP, management and treatment outcomes of DR-TB have remained sub-optimal. Reports from DR epidemiological surveys from different states in India have shown a high prevalence of MDR-TB in previously treated cases [6]. Several heterogeneous factors have mediated the success of DR-TB management in India. In this article, we discuss, in brief, some of the major challenges and implications associated with reducing the growing DR-TB epidemic in India.

## LOW RATE OF CASE NOTIFICATION AND LACK OF QUALITY PATIENT CARE IN THE PRIVATE SECTOR

**Figure 2.**
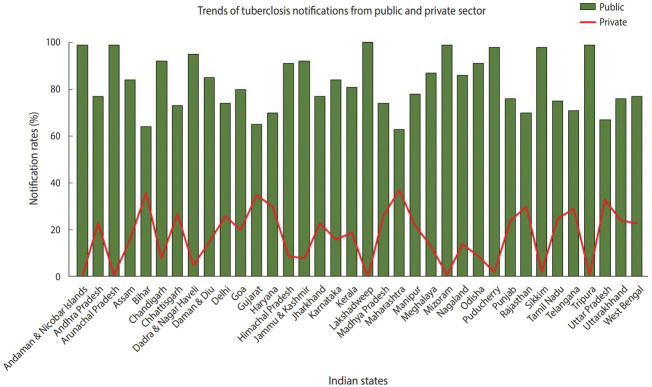
Notification rates (by percentage) from the public and private sectors in different Indian states. Source from: Arinaminpathy N, et al. Lancet Infect Dis 2016;16:1255-1260 [8].

TB is a notifiable disease in most countries. The term “notification” means that if a patient is diagnosed with TB, it is reported in the national surveillance system and to the WHO [7]. In 2012, the government of India made TB notification mandatory for the public and private sectors. In March 2018, the Ministry of Health and Family Welfare issued another order stating that doctors, pharmacists, chemists, and laboratory staff could face jail time if they fail to notify TB cases. Despite these stringent rules, India contributes approximately 25% of “missing” TB cases globally [2]. The WHO estimates that approximately 1 million TB cases in India are not recorded annually [1]. Notification data from different Indian states indicate that, while the proportion of case notifications from the public sector is consistently high, notifications from the private sector remain suboptimal (Figure 2) [8]. In India, diagnosis and treatment of TB in the public sector, such as in state-run hospitals, is low-cost or free-of-cost for TB cases that are reported. In contrast, TB care in the private sector, such as in private clinics and other private tertiary care hospitals, is not governed under the NTEP due to poor notification rates. State-issued health insurance is also not universally accepted by private hospitals in India. Despite high out-of-pocket expenditures and minimal insurance benefits, a large proportion of presumptive TB patients still seek and receive treatment from the private sector. A report from 2016 suggests that almost two-thirds of patients with a TB diagnosis annually seek care from the private sector in India [9]. In 2020 alone, around 34% of overall estimated TB cases were reported by the private sector—a staggering 40% increase from previous years [2]. In addition, around 50% of relapse cases notified by the public sector are treated in the private sector before reaching the NTEP [9,10].

One of the major reasons for poor treatment outcomes is a lack of quality control and the frequent use of non-NTEP approved therapy by private facilities. In 2017, under the revised NSP, the government of India launched the Joint Effort to Eliminate Tuberculosis, a globally-funded project to improve the quality of TB care patients receive in the private sector [2]. Despite significant efforts, the overall success of TB treatment in 2018 was only 35% in the private sector compared to 79% in the public sector [2]. Stronger political commitment and advocacy from policy-makers and community stakeholders for using WHO/nationally-endorsed guidelines for DR-TB care in the private sector will help improve treatment outcomes. In addition, further efforts are needed to increase the use of the national online TB notification portal “NIKSHAY” by private practitioners and health providers in urban, peri-urban, and rural areas to bolster DR–TB surveillance and notification rates from such regions.

## LIMITED DIAGNOSTIC CAPACITY FOR DR-TB IN PERIPHERAL AND RESOURCE-LIMITED REGIONS OF INDIA

Strengthening diagnostic capacity and rapid detection of DR-TB is crucial for the treatment of DR-TB cases in India. According to the WHO, for countries facing high rates of drug resistance, developing rapid detection tests and improving the management of patients with DR-TB is an urgent priority [5]. In India, the lack of rapid and prompt diagnoses in low-resource settings with high endemicity poses a major constraint on DR-TB treatment. As a result, it is estimated that around 56% of MDR-TB cases remain undiagnosed in India [11]. Mismanagement or delays in treatment initiation can also result in further transmission of DR strains in the community. In recent decades, there have been significant developments with respect to rapid molecular tests for diagnosing DR-TB. The list of WHO-endorsed molecular detection tests for TB and DR-TB can be found in Table 2. WHO-endorsed CBNAAT such as the GeneXpert/Xpert MTB/RIF assay (Cepheid, Sunnyvale, CA, USA) have been instrumental for diagnosing TB. GeneXpert is an automated real-time polymerase chain reaction (PCR) system and offers the advantage of simultaneous detection of MTB complex and rifampicin sensitivity in less than 2 hours, compared to conventional culture-based methods, which can take several days or weeks [12]. GeneXpert was included as a mainstay test in the U-DST guidelines by NTEP for high-risk groups to provide an appropriate regimen for TB patients. In India, although RR cases have been virtually regarded as a proxy for MDR-TB [12], high costs outside the public sector and reduced sensitivity of the assays in smear-negative cases represent major hurdles in providing accurate estimates of DR-TB and RR-TB in presumptive cases. Additionally, GeneXpert often fails to detect resistance outside the 81-bp rifampicin resistance determining region (RRDR) of the rpoB gene, thereby missing a sizable amount of cases with mutations at different sites [13].

To overcome issues associated with reduced sensitivity to the Xpert MTB/RIF assay, in 2017, the WHO recommended replacing existing Xpert MTB/RIF cartridges with a next-generation Xpert MTB/RIF assay (Xpert MTB/RIF Ultra) [14]. These Xpert Ultra cartridges include a larger chamber to allow a higher sample volume along with 2 MTB targets for higher diagnostic sensitivity in smear-negative cases. Results from multicenter studies comparing the diagnostic accuracy of Xpert MTB/RIF Ultra to Xpert MTB/RIF found that the sensitivity of the Ultra assay was 17% higher in smear-negative specimens and 12% higher in people living with human immunodeficiency virus, with a 3.2% lower specificity rate than Xpert MTB/RIF [15].

Alternatives to CBNAAT systems include commercial LPAs, such as GenotypeMTBDRplus (Hains Lifesciences, Nehren, Germany), which are also endorsed by the WHO and offer added advantages of increased sensitivity in addition to detection of MDR (isoniazid and rifampicin) in clinical samples [16]. However, their inapplicability for sputum smear-negative cases vastly overshadows their advantages as an effective diagnostic solution for MDRTB compared to CBNAAT. In 2017, the WHO introduced and recommended the use of rapid second line LPAs (MTBDRsl/Genotype MTBDRsl ver 2.0) for detecting DR to fluoroquinolones and other second-line drugs in confirmed MDR/RR cases [17]. Although the results of such tests are critical for placing patients on individualized oral regimens, they are mostly regarded as secondary tests to CBNAAT for confirming DR. Moreover, regardless of the added advantages and limitations of both CBNAAT and LPAs, their unavailability in the majority of diagnostic centers, peripheral laboratories, and in the huge private sector market has led to widespread diagnostic gaps and subsequently a low detection rate of MDR-TB cases in India.

**Figure 3.**
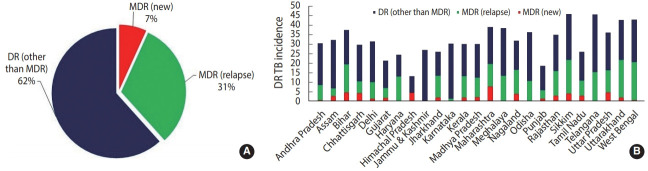
(A) overall and (B) state-specific incidence data of DR/MDR-TB in new and previously treated cases. DR other than MDR includes mono-resistance to either rifampicin or isoniazid. DR, drug resistant; MDR, multidrug-resistance; TB, tuberculosis. Source from: Central TB Division, Ministry of Health and Family Welfare. India TB report 2020: national tuberculosis elimination programme annual report [2].

To further boost the scalability and accessibility of CBNAAT systems for diagnosing DR-TB in peripheral zones, an indigenous point-of-care system (POC) called TrueNat^TM^ was introduced in India [18]. TrueNat^TM^ is an indigenous micro real-time PCR test developed by Molbio Diagnostics/Bigtec Labs, India, that offers greater sensitivity than conventional GeneXpert for diagnosing TB and detecting RR (TrueNat^TM^ MTB/MTB-RIF Dx). In addition, TrueNat^TM^ offers the added advantage of being used close to the POC, and it is battery-operated, making it particularly well-suited for use in low-resource primary healthcare settings with minimal infrastructure and training resources. In July 2020, the Indian Council of Medical Research announced that the WHO recommended the TrueNAT^TM^ platform as the frontline test for the initial diagnosis of TB and detection of RR [19]. While such developments have been promising and can greatly bolster DR-TB diagnosis, the current application of TrueNat^TM^ machines has been restricted to limited testing sites in only some Indian states. Additionally, one of the major critical gaps currently jeopardizing India’s TB elimination targets is the lack of diagnostic capacity of existing rapid CBNAAT assays for detecting DR to second-line drugs. This limitation is evident from national statistical data from 2019 suggesting a higher case rate of DR other than MDR (62%) (owing to large scalability) than MDR reported in new (7%) and previously treated cases (31%) (Figure 3) [2]. As a result, despite the enormous success of scaling up CBNAATs, just 44% of estimated MDR cases were diagnosed in India in 2019 [11]. In July 2020, the Foundation for Innovative New Diagnostics and Cepheid, Inc., announced the launch of the new Xpert MTB/XDR cartridges, which enable expanded DR profiling against multiple drugs such as isoniazid, ethionamide, fluoroquinolones, amikacin, kanamycin, and capreomycin with a turnaround time of less than 90 minutes [20]. While such tests can greatly overcome the diagnostic limitations of existing CBNAAT systems, they are still being evaluated by the WHO, and it may take considerable time to scale them up (with consideration of a cost-benefit analysis and infrastructure) after being introduced into the Indian market.

## CHALLENGES ASSOCIATED WITH TREATMENT ADHERENCE AND DISSEMINATION OF NEW TREATMENT GUIDELINES OF MDR-TB AMONG HEALTH PROVIDERS

Treatment adherence is crucial for successful TB control and eradication. However, the length and complexity of treatment protocols negatively impact patients’ adherence to those protocols and play a significant role in the emergence of DR-TB. Standard TB treatment regimens for new cases require patients to take a complex combination of drugs for 2 months in the intensive phase and 4 months in the continuation phase [5]. In the case of DR-TB, the treatment increases in duration to 24-48 months and entails a combination of second-line drugs and injections. These drug combinations, although potent, may have severe deliberating side effects that can restrict treatment adherence, lead to suboptimal treatment outcomes, and increase the risk of mortality and morbidity compared to treatment for drug-sensitive TB [21]. As a result, the treatment success rate for MDR-TB in India is around 48% compared to the global success rate of 56% [11]. Research on how to reduce drug toxicity and treatment duration has been an important goal in the WHO’s End Tuberculosis Strategy over the past 2 decades [1]. In 2012, 2 powerful, less toxic medicines, namely bedaquiline and delamanid, were released [22]. In 2018, the WHO released its new guidelines on the treatment of DR-TB, recommending a full oral regimen for MDR-TB patients. These guidelines were further updated, and new, consolidated guidelines were released in early 2020 that include a comprehensive set of WHO’s evidence-based policy recommendations on the treatment and management of DR-TB patients in low-income countries [23]. The new guidelines recommend a shorter-term (9-12 months) oral regimen with less focus on injections that includes categorical groupings of second-line drugs (A, B, C) for individualized treatment management according to the identified resistance. In India, current guidelines for DR-TB management are aligned with WHO recommendations and include U-DST (Xpert MTB/RIF and second-line LPA) for all presumptive cases and short-term MDR-TB regimens of 9-12 months [24,25]. The updated global guidelines provide the option of either using shortterm regimens including injections or individualized longer-term oral regimens using categorized drugs (including bedaquiline). While these changes look promising, India’s National TB program faces considerable challenges in terms of implementation, training, and monitoring adverse effects for nationwide dissemination. In addition, India has a huge private sector market and faces the difficult task of keeping private care providers up-to-date in their practices given the frequently changing global landscape for DR-TB management. In India, a large number of uninsured TB patients (missed by the national program) seek treatment from the private sector, which may not have up-to-date information regarding TB treatment or do not follow recent PMDT guidelines for DR-TB management. Moreover, while the MDR regimen is free of costs in the public sector, patients in the private sector are subject to enormous out-of-pocket treatment expenditures. In the public sector, various schemes have been rolled out under the revised NSP, which include payment of an incentive under a direct benefit transfer (DBT)scheme directly into the bank account of the beneficiary. Another DBT scheme is Nikshay Poshan Yojana (a nutritional supplement), which provides incentives to TB patients for nutritional support at the time of notification and subsequently through the treatment period. Other schemes include notification incentives to private care providers/informants for notifying TB cases through the NIKSHAY portal, transport incentives to support TB patients from tribal/remote regions, and honoraria to caretakers of TB patients for supporting TB patients [2].

In the private sector, while the government’s *Pradhan Mantri Jan Arogya Yojana* (Prime Minister’s scheme) provides limited insurance for inpatient therapy in economically weaker regions, outpatient costs are not covered, and incentives for TB-related hospitalization are poor. To reduce out-of-pocket expenditures in the private sector, the NTEP is pursuing mixed public-private ventures to optimize TB care through an initiative called Universal Access to Tuberculosis Care. While some pilot programs in a few states use free medicines from the NTEP, strict enforcement policies or guidelines have not been rolled out by the TB program regarding private providers’ use of free medicines, which can result in high, often catastrophic, costs to patients. Reports suggest that the average cost for treatment of pulmonary MDR and DR-TB in private healthcare settings ranges between US$ 5,000 and US$ 8,000, compared to US$ 50-100 for treatment for drug-sensitive TB, putting a catastrophic financial burden on patients’ families [26]. Lack of quality treatment and high costs in the unregulated private sector are critical reasons for low treatment adherence and poor treatment outcomes for MDR-TB in India.

Although updated treatment regimens will help to improve treatment adherence and also reduce the risks associated with prolonged drug intake, India will need a substantial upgrade to its TB health infrastructure to keep pace with the rapidly changing global landscape for DR-TB treatment and integrate those changes into national policy for rapid and timely dissemination to relevant health care providers.

## LACK OF STUDIES ON MOLECULAR EPIDEMIOLOGY AND TRANSMISSION DYNAMICS FOR MDR-TB IN HIGH ENDEMIC REGIONS

Molecular epidemiology studies are a critical resource for understanding the spread of TB, particularly MDR-TB, and its transmission dynamics. With an increase in the emergence of DR strains of TB, accelerated genomic studies are needed to determine the proportion of MDR-TB cases due to particular strains as opposed to ongoing transmission in rural and urban contexts. MTB complex genotyping methods have been widely used for investigating epidemics involving MDR-TB [27]. These tools are useful for determining the recent transmission factors of MDR-TB isolates and enable better control programs to be initiated to avoid MDRTB expansion of local or global population levels. India has a culturally diverse and heterogeneous population living in rural and urban areas. Thus, it is expected to host a genetically diverse set of *Mycobacterium tuberculosis* genotypes. In such population-based studies, isolates that share the same genotype are considered clusters and are assumed to be epidemiologically linked. Moreover, conventional phenotypic and WHO-endorsed rapid molecular testing methods such as Cepheid’s GeneXpert and Hains’ LPAs provide little or indirect information about drug susceptibility patterns, including a limited set of genetic mutation patterns associated with DR in a clinical sample. This information is vital for the management of DR-TB cases, especially with respect to the recent WHO-endorsed individualized short-term regimens. In an observational study carried out at P. D. Hinduja Hospital, Mumbai, among MDR-TB participants who were eligible for a short-term MDR regimen, > 5% were found to qualify based on clinical characteristics and DST results [28]. Among various genetic tools, next-generation sequencing (NGS) has emerged as a robust diagnostic method for the comprehensive characterization and detection of mutations in DR strains [29]. Unlike rapid molecular assays, NGS assays can provide extensive and detailed sequence information for multiple gene regions or whole genomes of interest. Such studies are highly important to public health, since they enable these programs to determine population-level risk factors for transmission to establish tailored public health strategies and gauge the success of control measures. Despite the advantages of NGS platforms, considerable challenges still exist for middle-income countries like India owing to cost limitations, the need for specialized well-trained staff, and the lack of readily-available data analysis and data storage solutions. In order to facilitate and accelerate genetic resistance prediction for DR-TB and to alleviate limitations associated with the analysis of whole-genome sequencing data, the “Comprehensive Resistance Prediction for Tuberculosis: an International Consortium” project (CRyPTIC) was launched in 2017. CRyPTIC is a collaborative initiative between the MRC Newton Fund, Wellcome Trust, and Bill and Melinda Gates Foundation that aims to provide comprehensive statistical solutions for the robust identification of genetic mutation patterns associated with DR strains worldwide [30]. The development of CRyPTIC is aligned with the WHO End Tuberculosis Strategy goals for providing rapid and tailored treatment strategies for DR patients via accurate genetic resistance prediction. The CRyPTIC project encourages public engagement and has developed relationships with several health-related institutions across 4 continents. In India, 2 Mumbai-based institutions, P. D. Hinduja Hospital and Medical Research Center and Foundation of Medical Research, are already a part of CRyPTIC. In the future, the participation of other public and private sector organizations in India in the CRyPTIC project will further help in rationalizing the drug regimen and bolstering treatment outcomes in MDR-TB cases.

## CONCLUSION

Despite the concerted efforts of the National TB program, the MDR-TB epidemic is on the rise in India. Integrated efforts are needed to bolster public-private partnerships to increase the notification rate of TB cases and improve the quality of care aimed at DR-TB patients. The scaling up of diagnostic capacity for both first-line and second-line drugs, complete adoption and rapid implementation of the 2020 WHO treatment guidelines in national policy, and the dissemination of those guidelines across the private sector will accelerate the efficacy of treatment and improve treatment outcomes. Identification of various genotypes of *M. tuberculosis* and studies on transmission dynamics to identify mediators of transmission for MDR-TB may be useful to determine the parameters for developing and boosting the programmatic management of DR-TB.

### Ethics statement

This paper is a perspective, so it did not need ethical approval.

## Figures and Tables

**Table 1.** Timeline depicting changes in India’s tuberculosis (TB) control policies

Timeline (year)	Actions/Achievements
1961	National tuberculosis program (NTP) launched by the government of India
1992	Joint review of NTP by government of India, World Health Organization (WHO), and Swedish International Developmental Agency
1993	NTP program renamed to Revised National Tuberculosis Control Programme (RNTCP) with implementation of WHO-endorsed Directly-observed Treatment Short Course (DOTS)
1993-97	RNTCP with DOTS (WHO) piloted in some states in India
1997	RNTCP with DOTS (WHO) launched nationwide by government of India
2002	Green light committee established to promote high-quality access to second-line drugs for appropriate use in TB control regimens
2006	Complete countrywide coverage achieved by RNTCP- DOTS for TB
	RNTCP become known as RNTCP-2
2007	RNTCP-2 launched programmatic management of drug-resistant tuberculosis (PMDT) to improve drug-resistant tuberculosis (DR-TB) care in India
2009	Rapid molecular tests and line probe assay (LPA) endorsed by WHO
2010	Cartridge-based nucleic acid amplification tests (CBNAAT) endorsed by WHO for rapid molecular diagnosis of TB and DR-TB
	Guidelines for PMDT released by government of India
2011	CBNAAT introduced in India
2012	National Strategic Plan for Tuberculosis (2012-2017) launched by the government of India
	The government of India prohibited sale of serodiagnostic kits and its use for TB diagnosis
	TB case notification made mandatory by the government of India
	Online TB case notification portal “NIKSHAY” launched by RNTCP-2 by government of India
2013	Complete geographic coverage for multidrug-resistant (MDR)-TB achieved by RNTCP-2
2014	Standards of TB care in India released containing universal guidelines for TB care and management for both private and public sectors
	First national drug resistance survey conducted by the government of India
2016	Technical and operational guidelines by RNTCP-2 for TB control in India (updated recommendations to standards for TB care in India) for use of short-term oral regimens and advisory for second line LPA for DR-TB
	MDR regimes with bedaquiline piloted in some states
2017	Revised National Strategic Plan (2017-25) launched by Prime Minister to end TB
	Decentralization of DR-TB services by implementation of guidelines of PMDT
	Launch of Joint Effort for Elimination of Tuberculosis program under revised NSP to improve quality of TB services in private sector and boost public private partnership
2018	WHO consolidated guidelines for DR-TB including use of short-term/full oral regimen for MDR-TB released and integrated under PMDT by RNTCP-2
2019	Implementation of TrueNAT as rapid molecular assay endorsed under RNTCP-2
	New guidelines for PMDT released based on WHO recommendation
2020	RNTCP-2 renamed as the National Tuberculosis Elimination Program
	The Indian Council of Medical Research endorsed TrueNAT based on WHO recommendation as mainstay test for universal drug susceptibility testing (DST) in India replacing conventional DST

**Table 2.** WHO-endorsed molecular diagnostic assays for the diagnosis of TB and drug resistance

Diagnostic test		Manufacturer	Platform/technology	Advantage	Limitation
CBNAAT					
	Commercial GeneXpert (Xpert MTB/RIF assay)	Cepheid, Sunnyvale, USA	Automated real-time PCR	WHO-endorsed front-line test for simultaneous detection of MTB complex and resistance to drug rifampicin in less than 2 hr	Expensive; Detection of mono-resistance; Reduced sensitivity in smear negative and paucibacillary samples
	Xpert^®^ MTB Ultra	Cepheid, Sunnyvale, USA	Automated real-time PCR	WHO-endorsed. ultra-large cartridge and additional MTB target for improved sensitivity for MTB detection in paucibacillary sample	Expensive; Detection of mono-resistance; Reduced specificity compared to GeneXpert cartridge
	Xpert^®^ MTB/XDR	Cepheid, Sunnyvale, USA and the Foundation for Innovative New Diagnostics, USA	Automated real-time PCR	Detection of MTB and drug resistance against multiple second-line drugs	Announced, under WHO evaluation; Not available for commercial use
Line probe assay					
	GenotypeMTBDRplus V1	Hains Lifesciences, Germany	Manual/Automated hybridization assay	WHO-endorsed assay for detection of MDR (resistance to frontline drugs isoniazid and rifampicin)	Requires culture or smear positive sample to rule out MDR
	GenoType MTBDRsl version 1.0	Hains Lifesciences, Germany	Manual/Automated hybridization assay	WHO-endorsed assay for ruling out MDR; Offers resistance detection to second line drugs	Requires culture or smear positive sample to rule out MDR
	GenoType MTBDRsl version 2.0	Hains Lifesciences, Germany	Manual/Automated hybridization assay	Offers resistance detection to second line drugs and injections	Reduced sensitivity in smear negative samples
	Genoscholar^TM^ NTM+MDRTB II	NIPRO, Japan	Manual/Automated hybridization assay	WHO-endorsed assay for differentiation of MTB species along with detection of MDR	Reduced sensitivity in smear negative samples
TrueNat^TM^					
	TrueNat^TM^ MTB	Molbio Diagnostics/ Bigtec Labs, India	Real-time microchip-based PCR system	Battery-operated point-of-care system for quantitative detection and diagnosis of MTB in 35 min	Reduced sensitivity in smear negative samples
	TrueNat^TM^ MTB Plus	Molbio Diagnostics/ Bigtec Labs, India	Real-time microchip-based PCR system	Quantitative detection of MTB and resistance to rifampicin in 35 min	Reduced sensitivity in smear negative samples
Commercial LAMP assay					
	Loopamp MTBC Detection Kit	Eiken Chemical Company Ltd., Japan	Manual point-of-care PCR assay	WHO-endorsed point-of-care PCR assay performed without thermal cycler; Results in less than 1 hr	Lower sensitivity than conventional PCR assays

WHO, World Health Organization; TB, tuberculosis; MTB, Mycobacterium tuberculosis; PCR, polymerase chain reaction; MDR, multidrug-resistance.
